# Perennial grassland led to more complex bacterial communities as well as inter-domain networks across three continuous monoculture systems

**DOI:** 10.3389/fmicb.2026.1823424

**Published:** 2026-05-21

**Authors:** Ruifen Zhu, Chang Liu, Bo Yao, Jishan Chen

**Affiliations:** Institute of Pratacultural Science, Chongqing Academy of Animal Sciences, Chongqing, China

**Keywords:** continuous monoculture, co-occurrence networks, inter-domain networks, microbial community, soil

## Abstract

Long-term continuous monoculture is a common practice in modern agricultural production, yet its effects on soil microbial communities—particularly the differences between annual crops and perennial forages—remain poorly understood. Here, we investigated the diversity, composition, and co-occurrence networks of soil bacterial and fungal communities under three long-term continuous monoculture systems in Northeast China: corn (annual crop, with fertilizer application), alfalfa (perennial legume forage, without fertilizer), and sheepgrass (perennial grass forage, without fertilizer). Fungal α- and β-diversity indices differed significantly across the three systems, whereas bacterial α-diversity showed no significant differences among them. The dominant bacterial phyla were Proteobacteria, Acidobacteria, Actinobacteria, and Gemmatimonadetes, while Ascomycota, Basidiomycota, and Glomeromycota were the most abundant fungal taxa. Co-occurrence network analysis revealed system-specific patterns: the bacterial network exhibited the highest number of nodes and edges in alfalfa, whereas the fungal network was most complex in sheepgrass. The ratio of positive to negative edges in the bacterial network was more than three times higher in alfalfa than in the other systems, while the fungal network showed the highest positive-to-negative link ratio in sheepgrass. Inter-domain associations were predominantly characterized by a higher number of edges in sheepgrass. Our findings demonstrate that perennial forage systems (alfalfa and sheepgrass) promote more complex bacterial and inter-domain microbial networks compared to the annual corn system. These differences in network may have important implications for soil ecosystem functions mediated by microbial communities, highlighting the potential benefits of incorporating perennial forages into long-term monoculture systems to enhance soil microbial interactions and ecosystem sustainability.

## 1 Introduction

Crop yield and quality are key to the success of farming systems (Sala et al., 2015), and continuous monoculture is a common system of modern intensive agricultural production to improve crop yield and quality (Zabel et al., 2019; Allan et al., 2023). However, long-term continuous monoculture will speed up the consumption of key nutrients, and lead to their loss to the environment and, subsequently, deterioration of soil physical and chemical properties, as well as soil microbial community changes. It has been reported that long-term continuous monoculture can decrease soil pH, organic matter and available potassium, while increasing total nitrogen and available phosphorus (Burney et al., 2010; Cameron et al., 2013; De Deyn and Lammert, 2021; Wardle and Lindahl, 2014) and inhibiting the mineralization of carbon and nitrogen (Hartmann et al., 2015), thus hindering the nutrient cycling in the soil. However, little is known about the effect of long-term continuous monoculture on soil microbiome networks.

Soil microorganisms play a key role in nutrient cycling, organic matter decomposition, and ecosystem functioning. Bacteria and fungi, as major components of the soil microbiome, are jointly involved in the cycling of carbon, nitrogen, and phosphorus. Among them, soil bacteria are highly diverse (Hartmann et al., 2015) and play an important ecological role in the transformation and cycling of soil nutrients and organic matter (Lauber et al., 2008). The composition and functional structure of soil bacterial community are closely related to the cycling of carbon, nitrogen and phosphorus and the decomposition of organic matter (Lef et al., 2015; Li et al., 2019; Turley et al., 2020). Due to the rapid response of soil bacteria to environmental changes, soil bacteria are considered as effective biological indicators of soil fertility and soil management (Philippot et al., 2013a; Philippot et al., 2013b). Soil bacterial community diversity and structural changes have been shown to be associated with plant species, land use types and soil physicochemical properties (Banerjee et al., 2024; Wagg et al., 2020; Banerjee et al., 2019). The soil environment generally shapes the composition, diversity, interaction pattern and functional structure of soil bacterial community (Estendorfer et al., 2017; Hartman et al., 2018; Tardy et al., 2022; de Vos et al., 2017; Pérez-Valera et al., 2017), and soil bacteria also rely on their metabolic activity to create a suitable soil environment for their survival (Friedman et al., 2017; Geisen et al., 2019), thereby improving soil fertility. Bacteria have complex interactions in the soil and are involved in forming ecological networks and maintaining system functions (Russel et al., 2017). Ecological interaction networks have provided new insights into the interplay between soil bacterial communities, enabling a more comprehensive understanding of bacterial community structure and the ecological rules that guide community assembly (Tardy et al., 2022). It has been reported that the molecular ecological network of soil bacterial community will become more complex and increase in modularity and the number of modules as plants grow (Gao et al., 2019; Geisen et al., 2018). Although numerous studies have shown that soil physicochemical properties affect bacterial community structure, research on the relationship between these properties and the topological characteristics of bacterial co-occurrence networks remains relatively limited. As an important member of the soil microflora, soil fungi, together with other microorganisms, participate in the material cycle and energy flow in the soil ecosystem (Xiong et al., 2020; Rüger et al., 2023). It has been found that fungal communities can affect the diversity and composition of terrestrial plant communities through reciprocal symbiosis, disease and nutrient uptake and cycling (Clarholm and Skyllberg, 2013). In addition, soil fungi can affect plant physiological status through biological processes such as nitrogen fixation, plant hormone secretion, pathogen protection, and enhanced plant drought resistance (Ortiz-Alvarez et al., 2018; Wagg et al., 2019). However, the response of soil fungal communities to long-term continuous monoculture, particularly of forage species, remains poorly understood. Therefore, their co-variation and co-occurrence networks under long-term continuous monoculture remain poorly understood.

Co-occurrence networks are often used to explain relationships between microorganisms (Hu and Chabbi, 2021). Although co-occurrence patterns may not reflect the true co-occurrence networks of microbial interactions, this integrated approach has contributed to a better understanding of the impact of continuous monoculture on microbial communities (Hu and Chabbi, 2021; Fer et al., 2020). Network analysis can also reveal why some microbial communities always appear together, or whether certain microbial taxa are more important for maintaining network structure. The co-occurrence networks can be assessed by associated network scores-such as degree, clustering coefficient, and modularity-which indicate connectivity between operational taxonomic units (OTUs). Recent studies have shown that network connectivity can have important implications for microbiome stability (Pester et al., 2014).

Here, we evaluated the response of soil microbial community to long-term continuous monoculture by examining how the diversity, composition and co-occurrence networks of soil microbiomes differ between corn (a typical annual crop with fertilizer application) and forage plots (perennial grasses without fertilizer application) under continuous monoculture systems. While the effects of plant species and fertilization on soil microorganisms have been extensively studied individually, how these factors interact to shape soil microbial communities under long-term continuous monoculture remains unclear. Specifically, by leveraging a long-term (>10 years) replicated field experiment that includes two continuous monoculture systems (corn and forage), we aimed to compare the relative contributions of plant life form (annual vs. perennial) and management practice (fertilized vs. unfertilized) to soil microbial diversity, community composition, and co-occurrence network. In each system, we measured a range of soil physical, chemical, and microbial properties. We also quantified the diversity and composition of soil bacteria and fungi and constructed co-occurrence networks to explore the associations among microbes.

## 2 Materials and methods

### 2.1 Experimental design and soil sampling

Soil samples were collected from a long-term field experiment at the Frigid Forage Research Station. The station has an altitude of 160 m, longitude of 125° 28′ 24″ E, latitude of 46° 32′ 17″ N in Harbin City, Heilongjiang Province, China. The experiment site occupies a sub-humid climate and a dark loam (mostly chernozem, FAO taxonomy) (Bower et al., 2004). The soil is a Cambisol with loamy texture. Soil chemical properties were shown in Supplementary Table S1.

The experiment was designed to investigate the effects of long-term continuous monoculture systems on soil microbial communities and their co-occurrence patterns.

The experiment was established in 2010 in a randomized complete block design divided in four blocks, each containing plots with a surface of 6,700 m^2^. The dominant varieties were corn (*Zea mays* L., an annual plant), alfalfa (*Medicago sativa*, a perennial legume), and sheepgrass [*Leymus chinensis* (Trin.) Tzvel., a perennial grass]. Each containing three long-term continuous monoculture systems established: corn (served as a control), alfalfa, and sheepgrass. In these systems, conventional tillage and fertilizer were applied only in the corn field, whereas in the alfalfa and sheepgrass grasslands, no fertilizer was applied and mowing management was guided by a mowing index that provides the optimum cutting times for potential plant production.

Sampling was conducted in September 2019. In each replicate plot, five soil cores (20 cm depth, 5 cm diameter) were collected randomly and pooled to form one composite sample. With 20 replicate plots per treatment, a total of 20 composite samples were obtained for each treatment (*N* total = 60 samples). Plant debris and roots were removed from soil samples, which were then homogenized and sieved at 4 mm. Soil relative moisture was determined by drying 10 g of fresh soil at 105 °C for 24 h.

### 2.2 Assessment of microbial community composition and diversity

To characterize the soil bacterial and fungal communities under three long-term continuous monoculture systems (corn, alfalfa, and sheepgrass), we performed DNA metabarcoding targeting the 16S rRNA gene and the fungal ITS1 region. DNA was extracted from all soil samples using the DNeasy PowerSoil-htp 96 well DNA isolation kit (Qiagen, France), and Illumina sequencing was conducted to analyze microbial diversity and composition. For all DNA extracts, there were two steps. Firstly, the V3–V4 hypervariable region of the bacterial 16S rRNA gene was amplified by polymerase chain reaction (PCR) using the fusion primers U341F (5′-CCTACGGGRSGCAGCAG-3′) and 805R (5′-GACTACCAGGGTATCTAAT-3′) (Simon et al., 2015). Secondly, fungal ITS1 region was amplified using the primers ITS1F (CTTGGTCATTTAGAGGAAGTAA) and ITS2 (GCT GCGTTCTTCATCGATGC) primers (Gardes and Bruns, 1993; White et al., 1990). Sequencing was performed on MiSeq (Illumina, 2 × 250 bp and 2 × 300 bp) using the MiSeq reagent kit v2 and v3 (500 and 600 cycles, respectively).

### 2.3 Sequencing and bioinformatic analysis

Sequence data from the soil samples was analyzed using an in-house developed Python pipeline.^1^ Briefly, 16S rRNA gene and ITS sequences were assembled using PEAR (Zhang et al., 2014) with default settings. Further quality checks were conducted using the QIIME 1 pipeline (Caporaso et al., 2010) and short sequences were removed (<400 bp for 16S and <300 bp for ITS). The obtained sequences and the sequences downloaded from GenBank were used to generate the phylogenetic tree using the unweighted average method (UPGMA) of Mega5.0, and the Bootstrap method was used to test the phylogenetic tree. The Bootstrap dataset is 1,000 times. Reference based and *de novo* chimera detection, as well as OTU clustering were performed using VSEARCH (Rognes et al., 2016) and the adequate reference databases (SILVA (138.1) representative set of sequences for 16S, and UNITE (8.0)’s reference dynamic dataset for ITS). For 16S rRNA, taxonomy was assigned using UCLUST (Edgar, 2010). For ITS, the taxonomy assignment was performed using BLAST (Quast et al., 2013) and the UNITE reference database v.7-08/2016 (Guillou et al., 2013). Raw sequences were deposited at the NCBI under the BioProjects PRJNA973260: CMS2023, https://www.ncbi.nlm.nih.gov/bioproject.

Based on taxonomic assignments, we filtered out operational taxonomic units (OTUs). In total, 2,319,835 bacteria sequences and 3,244,474 fungal sequences were obtained and assigned to 6,999 and 1866 OTUs, respectively. Bacterial and fungal α-diversity metrics (i.e., observed species, Simpson’s reciprocal, Shannon as well as Faith’s Phylogenetic Diversity PD for bacteria) (Guillou et al., 2013) were calculated based on rarefied OTU tables (23,000 sequences per sample for 16S rRNA and 35,000 sequences per sample for ITS). Bray–Curtis dissimilarity matrix were also computed to detect variations in the structure of microbial communities.

### 2.4 Statistical analyses

Statistical analyses were conducted using R statistical software version 3.4.1 (Muyzer et al., 1993). Differences in gene copy number (16S rRNA, ITS) were tested using the Kruskal–Wallis test followed by Dunn’s multiple comparison test (adjusted *p*-value <0.05). Differences in the microbial α-diversity indices were tested using ANOVA followed by Tukey’s honestly significant difference (HSD) test (*p*-value <0.05) using the agricolae package (Bru et al., 2011). To explore the relationships between soil physicochemical properties and microbial community structure, redundancy analysis (RDA) was performed using the vegan package in R. Mantel tests were used to assess correlations between Bray–Curtis dissimilarity matrices of microbial communities and Euclidean distance matrices of soil properties.

Normality and homogeneity of the distribution of residuals were verified and log-transformations were performed when necessary. Non-metric multidimensional scaling (NMDS) was performed based on the Bray–Curtis dissimilarity matrices to visualize community composition patterns across treatments, and stress values were calculated to evaluate the ordination fit. Permutational multivariate analysis of variance (PERMANOVA) was carried out on the Bray–Curtis dissimilarity distance matrices using “adonis” function implemented in the vegan package (Leininger et al., 2006). Pairwise *post-hoc* tests were conducted using the function “pairwise.adonis” from the pairwiseAdonis package (Poly et al., 2001) with Holm corrections. As sequencing data are usually sparse, low abundance OTUs were filtered out by keeping OTUs that are present at a threshold of 0.04% in all samples. We also discarded the OTUs that were not found in at least 10 out of the 60 samples. This filtering step allows reducing the zero counts in sequencing datasets, which can inflate the number of false positive for the differential abundances analysis and spurious correlation between OTUs in network analysis. This resulted in 472 OTUs for 16S rRNA and 218 for ITS. Differential abundance analysis of microbial community composition was conducted by pairwise comparisons between land uses of filtered count matrices (*n* = 20) using the DESeq2 package (FDR-corrected *p*-value <0.00001) (Mohan et al., 2004). Significantly discriminant OTUs were illustrated by ternary plots using ggtern package (Welsh et al., 2014).

All networks were constructed using filtered count matrices (i.e., 472 OTUs for 16S rRNA, and 218 for ITS). Bacterial and fungal networks were inferred separately for each long-term continuous monoculture system and for visualization purpose, and only partial correlations with |*ρ*| > 0.1 were considered. Inter-domain networks were inferred using all microbial groups for each system, and for visualization purpose partial correlations with |ρ| > 0.08 were visualized. Networks were then visualized using the Cytoscape software (R Core Team, 2020). The Network Analyser tool from Cytoscape was used to calculate network topological parameters (i.e., nodes, edges, clustering coefficient and degree).

We inferred microbial association networks from the soil samples of the three systems for the bacterial and fungal communities using a recently developed sparse multivariate Poisson log-normal model. The networks were estimated using network size [i.e., num.vertices (*n*), num.edges (*L*)], ratio of the positive to negative num.edges in the networks, the clustering coefficient (Average.CC, i.e., the degree to which nodes are clustered) and the average degree (Average K, i.e., average edges per node) (Supplementary Table S2).

## 3 Results

### 3.1 Long-term continuous monoculture impacts soil microbial communities

Significant differences in α-diversity indices (Richness, Shannon_index, Chao1and Ace) were observed for the bacterial community, only with Shannon index being higher in continuous monoculture of alfalfa and sheepgrass than in corn (Tukey’s test, *p*-value<0.05, Supplementary Figures S1, S2). By contrast, the α-diversity indices of the fungal communities were significantly different across the three systems.

**Figure 1:**
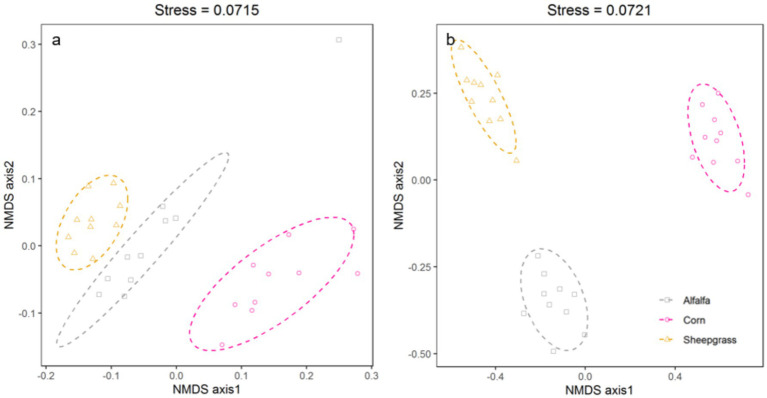
Non-metric multidimensional scale (NMDS) of bacterial **(a)** 16S rRNA gene fragments and fungal **(b)** ITS1 amplicons showing shifts in community structure three continuous monoculture systems.

Non-metric multidimensional scale (NMDS) results with stress 0.0715 and 0.0721 for bacterial and fungal communities, respectively, also showed the same trends among the communities in the three systems (Figure 1). Based on NMDS, soil bacterial and fungal communities of alfalfa and sheepgrass were significantly different from those of corn. Using PERMANOVA of microbial community structure, the results for soil bacteria community were *R* = 0.7041, *p*-value = 0.001 < 0.05, while PERMANOVA for soil fungi community was *R* = 0.9757, *p*-value = 0.001 < 0.05. In brief, the results of ANOSIM demonstrated the communities in these systems were significantly different. However, the structure of the bacterial communities was more similar between continuous monoculture of alfalfa and sheepgrass than continuous monoculture of corn from non-metric multidimensional scale (NMDS). Soil physicochemical properties differed among the three systems (Supplementary Table S1). Specifically, alfalfa and sheepgrass, which received no fertilizer, exhibited higher OM and AN than corn.

RDA revealed that OM, AN, and pH were the most significant factors explaining variation in bacterial community structure (*p* < 0.001), accounting for 57.02 and 13.84% of the total variation on the first two axes. For fungal communities, OM, TN, and AK were the primary drivers (*p* < 0.001), explaining 42.77 and 23.81% of the variation. Mantel tests confirmed significant correlations between microbial community dissimilarity and soil property dissimilarity (bacteria: *r* = 0.331, *p* < 0.001; fungi: *r* = 0.579, *p* < 0.001) (Supplementary Figure S3).

### 3.2 Identifying differentially abundant OTUs

**Figure 2:**
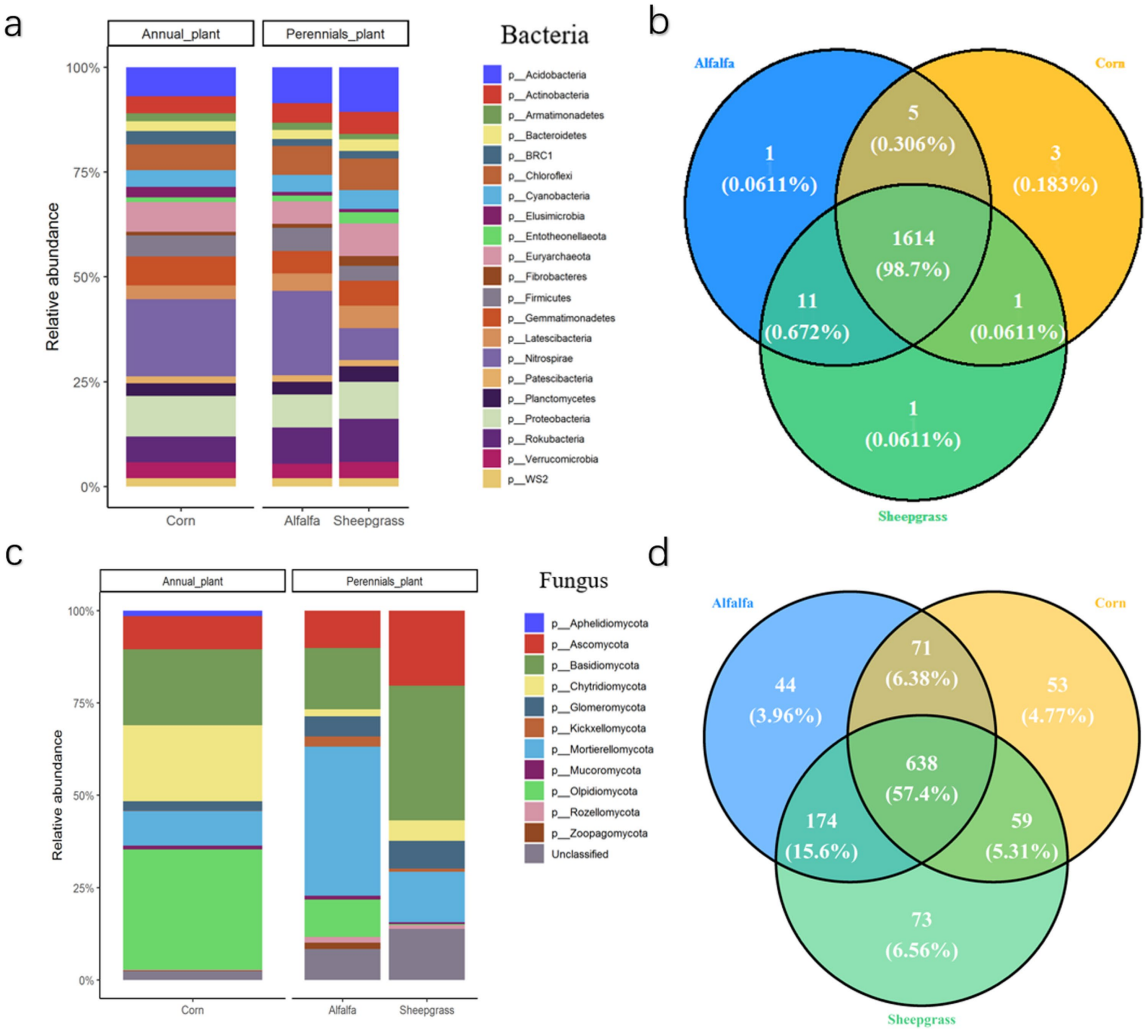
Abundances of total bacteria (16S rRNA gene) and fungal (ITS) among the three systems. Relative abundance at the phylum level in the total bacterial **(a)** fungal **(c)** community. The Venn diagram represents the number of common and unique OTUs in bacterial **(b)** fungal **(d)** community, and can compare the similarity and overlap of OTUs composition of environmental samples. Different colors represent different samples.

Across all samples, Proteobacteria was the most abundant bacterial phylum, accounting for 25.1, 25.0, and 24.9% of bacterial sequences in corn, alfalfa, and sheepgrass treatments, respectively; Actinobacteria accounted for 11.3, 11.2, and 11.3%; and Gemmatimonadetes accounted for 9.8, 9.9, and 9.9%. In fungi, Ascomycota were of 37% in alfalfa, 40% in corn and 39% in sheepgrass. Basidiomycota were of 10% in alfalfa, 10% in corn and 11% in sheepgrass. Glomeromycota were of 4% in alfalfa, 4% in corn and 3% in sheepgrass. Those represented the most abundant fungal taxa (Figure 2).

The Venn plots showed the distribution of the most abundant OTUs. Differential abundance analysis, based on the most abundant OTUs (1,636 OTUs for 16S rRNA and 1,112 for ITS), identified 22 bacterial OTUs with significant changes in abundances among three systems (FDR-corrected *p* < 0.00001) (Figure 2a). The strongest differences were observed among systems, with 3 discriminant bacterial OTUs being significantly more abundant under continuous monoculture of corn (Figure 2b). By contrast, only one OTU showed a significant difference between the alfalfa and sheepgrass systems. OTUs affiliated with Acidobacteria and Gemmatimonadetes were the most responsive to the three systems, exhibiting modest changes in relative abundance across treatments (Figure 2b).

For the fungal community, 474 out of 1,112 fungal OTUs were significantly impacted by the three systems (Figure 2c). Different patterns were observed for bacteria, and the largest differences were between the continuous monoculture of sheepgrass and the continuous monoculture of corn, with 73 OTUs being more abundant under sheepgrass (e.g., Ascomycota and Basidiomycota), and 53 OTUs mainly assigned to Glomeromycota being more abundant under continuous monoculture of corn (Figure 2d). We found that only 20 OTUs belonging to fungi significantly increased under sheepgrass, while 9 OTUs that belonged to all three domains decreased under alfalfa.

### 3.3 Co-occurrence networks within microbial groups were influenced by the long-term continuous monoculture systems

**Figure 3:**
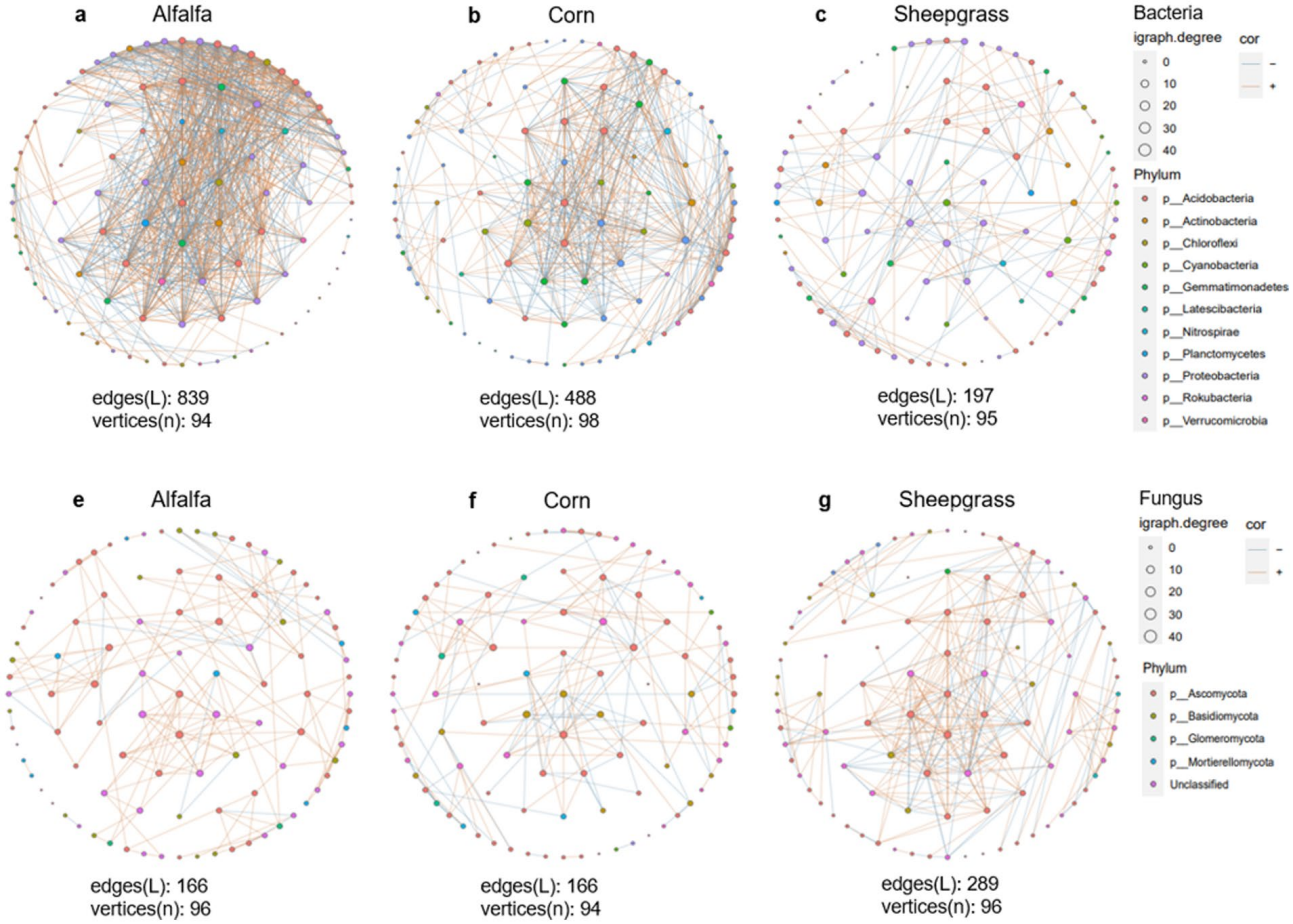
Co-occurrence networks of **(a,c,e)** bacterial and **(b,d,f)** fungal communities in three continuous monoculture systems. Nodes are colored according to their taxonomic affiliation at phylum levels. The size of the nodes is proportional to the number of edges per node. Link thickness is proportional to partial correlations between nodes and represents associative (blue, *ρ* > 0.1) or exclusionary relationships (yellow, *ρ* < −0.1). The networks was estimated using network size [i.e., num.vertices (*n*), num.edges (*L*)]. The lowercase letters in the upper left corner (in bold) are each network **(a–f)**.

In the bacterial and fungal networks, both the number of nodes and edges were significantly different among the three systems (Figure 3 and Supplementary Table S2). Alfalfa has the largest number of nodes and edges in the bacterial network (Figures 3a–c) and sheepgrass has the largest number of nodes and edges in the fungal network (Figures 3d–f). However, the highest ratio of positive to negative edges in alfalfa was more than three times higher than that in the others (alfalfa = 6.22, corn = 1.48 and sheepgrass = 2.04). The fungal networks showed a different trend with a higher ratio of positive to negative edges in sheepgrass than in corn. The fungal network also revealed an increase in the ratio of positive to negative edges in alfalfa (6.22), similar to bacterial networks. Networks from the alfalfa of the bacterial communities exhibited the highest clustering coefficient and it was similar to sheepgrass of the fungal communities. Similarly, the average clustering coefficient is also the highest in networks from alfalfa of the bacterial communities and networks from sheepgrass of the fungal communities (Supplementary Table S2).

### 3.4 Associations across microbial groups differ among the long-term continuous monoculture systems

To better understand how plant species and management influences associations between bacteria and fungi, we inferred cooccurrence networks including all two groups for each system (Supplementary Figure S4). The microbial network with two groups from alfalfa (Supplementary Figure S4a) was the most complex with 1781 nodes and 804 edges, followed by corn (Supplementary Figure S4b) with 1,033 nodes and 570 edges, and then the network from sheepgrass (Supplementary Figure S4c) with 522 nodes and 498 edges. The same is true that we found a higher and average degree and average.path.length in network from alfalfa indicating that the nodes were more resistant to environmental interference than those of other systems. Clustering coefficient indicated that the network from sheepgrass was more sensitive and responsive to external environmental factors.

**Figure 4:**
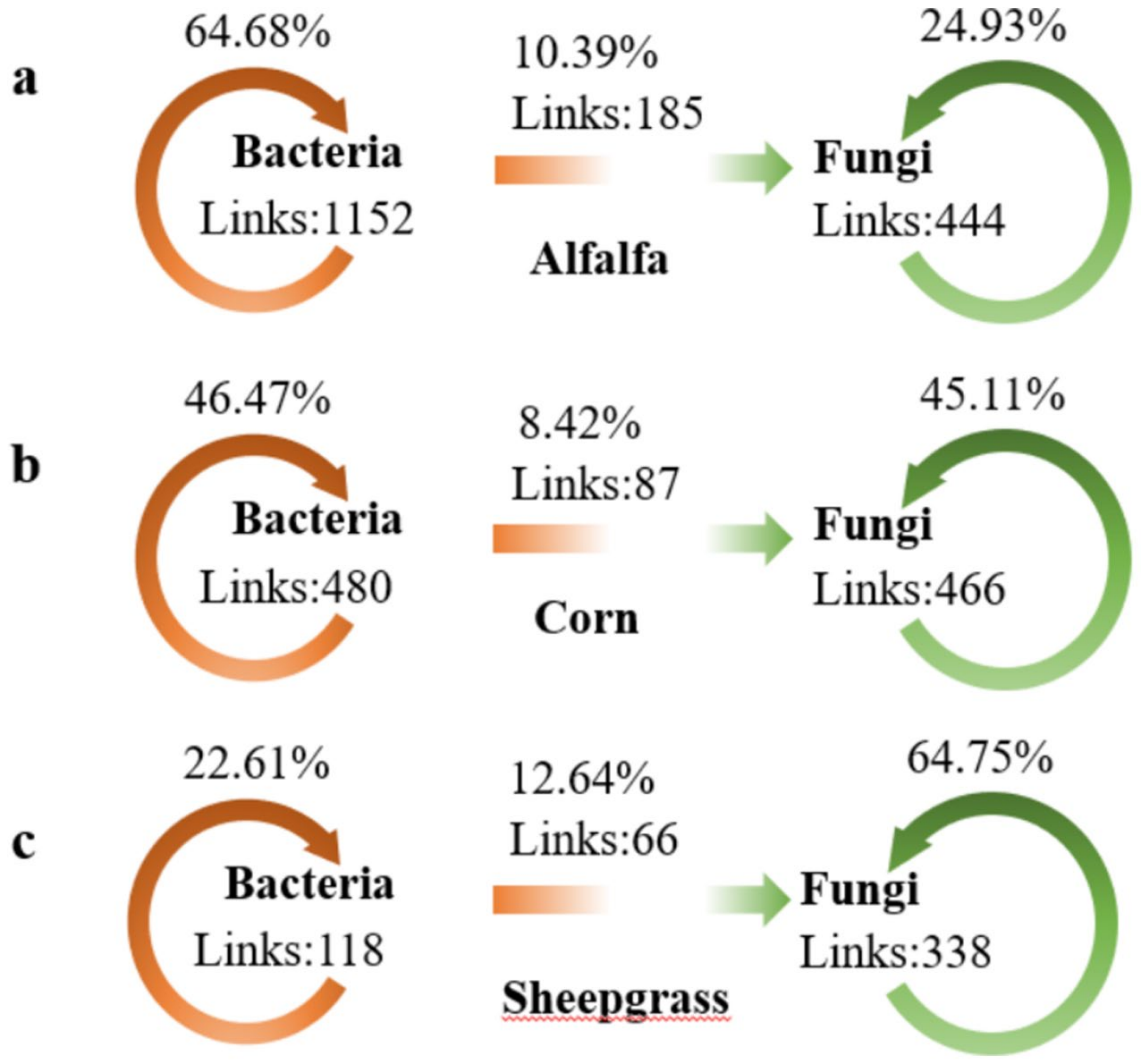
The number of positive and negative edges within and between microbial groups. Microbial networks were dominated by bacteria or fungi associations. This was also the case for bacteria-fungi associations **(a–c)**.

In contrast, the comparison between inter-group networks showed that 32.6% of nodes were shared among the three systems. Only one microbial link (bacteria–fungi) was shared between to the networks from the three systems (Figure 4). To distinguish differences in taxa co-occurring among the systems, we compared the number of positive and negative edges within and between microbial groups. Regardless of system, microbial networks were dominated by bacteria–fungi associations (10.39%) for alfalfa (Figure 4a), bacteria–fungi associations (8.42%) for corn (Figure 4b), and bacteria-fungi associations (12.64%) for sheepgrass (Figure 4c). Bacteria–bacteria associations represented 64.68% from alfalfa, 46.47% from corn and 22.61% from sheepgrass of the total number of edges, respectively. In contrast, fungi–fungi associations represented 24.93, 45.11 and 64.75% of the total number of edges, respectively. However, the positive to negative link ratio for the bacteria-bacteria associations was the highest in sheepgrass (alfalfa = 1.89, corn = 3.27 and sheepgrass = 5.37). Associations across the two groups were mainly characterized by a higher number of edges in sheepgrass (perennial grasslands). This was also the case for bacteria–fungi associations (Figures 4a–c).

## 4 Discussion

Studies of long-term continuous monoculture systems and their belowground effects have focused primarily on bacterial and/or fungal communities. However, fungi and bacteria interact through various mechanisms, including competition, mutualism, and antagonism, which together shape soil microbial community structure and function (Gao et al., 2019; Mirza et al., 2020), underscoring the need to adopt holistic approaches for a comprehensive understanding of the soil microbiome (Geisen et al., 2019). Here, we compared the soil bacterial and fungal communities in three long-term continuous monoculture systems over 10 years of experimentation. We found that β-diversity of all three systems was affected by plant species, with significant differences among alfalfa, corn and sheepgrass. Little is known about the importance of long-term continuous monoculture systems in driving soil bacteria-fungi associations and there are large discrepancies between studies (Geisen et al., 2018). Although climatic factors have been identified as the top predictor of composition of fungal communities (Zhou et al., 2023), our results indicate that fungi can be affected by changes in these systems to the same extent as bacterial communities. However, the α-diversity of the fungal communities showed significant differences across the three systems, although the bacterial α-diversity did not show significant differences, with the highest diversity in continuous monoculture with alfalfa, corn and sheepgrass. Similarly, previous studies found that shifts to agricultural land-use increased bacterial diversity (Geisen et al., 2023).

It is likely that perennial plant communities in these systems have led to a greater diversity in soil nutrients through root exudates and crop residues, leading eventually to increased bacterial diversity (Zhao et al., 2019). Among the three systems were associated with shifts in soil properties, perennial forages (alfalfa, sheepgrass) showed higher SOM and available nitrogen than corn, which correlated with greater network. Alfalfa’s nitrogen fixation likely enhanced bacterial networks, while sheepgrass’s root carbon inputs favored fungal networks. These findings suggest perennial forages promote cooperative microbial interactions through improved soil properties. These properties were positively correlated with the co-occurrence networks of bacterial and fungal, suggesting that the more complex microbial networks observed in perennial forage systems were at least partially mediated by enhanced soil organic matter and nitrogen availability. This is consistent with the observed differences in the biochemical nature of the soil organic matter (SOM) between the long-term grasslands and continuous monoculture systems studied here (Crème et al., 2018). The relative abundances of N-effective nutrient (i.e., N, AN) were the highest in the alfalfa and sheepgrass systems, suggesting that plant species can be a driver for these functional groups. However, since microbial communities involved in N-cycling processes could result in nitrogen losses, additional work is needed to further determine the fate of inorganic N in these systems.

Considering the differences in eco-evolutionary dynamics between perennial and annual systems, it can be hypothesized that plant–soil feedbacks can lead to more complex and connected networks in these systems. However, when inferring microbial cooccurrence networks for each group (i.e., bacteria and fungi), the bacterial network in terms of the number of nodes and edges was more complex in alfalfa (perennial forage) than in corn and sheepgrass. At the same time, the fungi network in terms of the number of nodes and edges was more complex in sheepgrass (perennial forage) than in corn and alfalfa. This result agrees with a previous study that showed higher bacterial network in perennial grassland than in annual cropping systems (Karimi et al., 2019).

Interestingly, soil bacterial diversity was also not significantly different between perennial forage varieties and corn, suggesting that changes in soil bacterial diversity do not necessarily reflect changes in microbial networks (He et al., 2020). In contrast to a previous study showing that agricultural intensification reduces fungal network (Banerjee et al., 2019), we found a higher α-diversity in sheepgrass than in other systems. This discrepancy might be explained by the difference root associated fungi were monitored in the previous study (Banerjee et al., 2019) whereas the present study focused on soil communities. Differences in the structure and composition of the fungal community across plant species were mirrored in co-occurrence networks. Specifically, when the link type was taken into consideration, an increase in the positive to negative link ratio in sheepgrass for fungi as well as in the other groups was found, indicating that the positive associations between soil microorganisms were promoted. This pattern might be owing to increased niche differentiation when comparing different plant species, which resulted in increased collaboration between microbial species. On the contrary, the increase in the positive to negative link ratio in these systems suggests more effective cooperation was established rather than that the competition between microbial taxa increased. Alternatively, previous work showed that differences in community evenness likely affect the positive edge percentage since fewer prevalent taxa tend to contribute more to negative edges (Karimi et al., 2019; Morrien et al., 2017). In any case, further studies are required to elucidate these changes in co-occurrence networks among the three systems.

Finally, we inferred networks encompassing all three groups to obtain an integrated and holistic view of the soil microbiome. Different from the corn, we found a higher in the inter-group network under alfalfa and sheepgrass. This finding supports our hypothesis that perennial grassland lead to more connected inter-group microbial networks. This increase in co-occurrence networks was likely due to an increase in the number of bacterial and fungal nodes as well as bacteria-fungi edges. While almost half of the nodes of the inter-domain networks were similar, edges were distinct among the three systems. That plant species and management practices affected associations within the soil microbiome suggests that land-use type might be an important factor and should be considered in future management, given that recent studies have shown that ecosystem functioning could be related to microbial co-occurrence networks (Wagg et al., 2019; Morrien et al., 2017).

Despite the different systems studied, bacteria-fungi were dominant in soil microbiome network, especially the sheepgrass microbial networks, which was highly connected to both bacteria and fungi. Although co-occurrence networks suffer spurious correlations when the effects of habitat filtering are strong, they can also recapitulate possible interactions between microorganisms under certain conditions (Zhao et al., 2019; Berry and Widder, 2014). However, we found more negative associations between bacteria and fungi. Interestingly, recent work suggests that protist feeding on fungi might be equally important (Geisen et al., 2023). On the other hand, some fungi have developed trapping structures, such as adhesive spores and hyphae, to capture soil-inhabiting microorganisms, such as protists (Corsaro et al., 2018). Fungi can also be protist parasites, when, for example, amoeba ingests the spores or conidia and kills it (Scheid, 2018). These predatory/parasitic interactions might explain some of negative associations observed in our study, however, this study lacks research on soil protozoa.

## 5 Conclusion

Under three long-term continuous monoculture systems (corn, alfalfa, and sheepgrass), soil microbial communities exhibited distinct shifts in composition and network structure. Fungal α- and β-diversity differed significantly across systems, while bacterial α-diversity did not. Co-occurrence networks showed system-specific patterns: bacterial networks were most complex in alfalfa, and fungal networks in sheepgrass, with both perennial forages showing higher positive-to-negative edge ratios than corn. These differences were associated with higher soil organic matter and available nitrogen in the perennial systems. Collectively, our findings suggest that perennial forages promote more complex and cooperative soil microbial networks, highlighting their potential to enhance soil health in long-term monoculture systems.

## Data Availability

The datasets presented in this study can be found in online repositories. The names of the repository/repositories and accession number(s) can be found at: https://www.ncbi.nlm.nih.gov/, PRJNA973260.

## Footnotes

1
https://forgemia.inra.fr/vasa/illuminametabarcoding
