# Supplementary material for: Perennial grassland led to more complex bacterial communities as well as inter-domain networks across three continuous monoculture systems
Source: Front Microbiol. 2026 May 21;17:1823424. doi: 10.3389/fmicb.2026.1823424 (PMC13235987; doi:10.3389/fmicb.2026.1823424)
Supplement: Supplementary file 1 [file Supplementary_file_1.DOCX]

Additional file 1

Table S1 The chemical properties of soil in the three different treatments. The letters in each column indicate significant differences (P<0.05).

|  | N (g/kg) | P (g/kg) | K (g/kg) | AN (g/kg) | AK (g/kg) | AP (g/kg) | OM (g/kg) | pH |
| --- | --- | --- | --- | --- | --- | --- | --- | --- |
| Corn | 0.13 b | 0.03 c | 0.14 c | 0.05 c | 0.21a | 0.05 c | 23.25c | 6.11c |
| Alfalfa | 2.75 a | 1.69a | 2.15 a | 0.17 a | 0.22a | 0.14 a | 33.25b | 7.02a |
| Sheepgrass | 2.46 a | 0.39 b | 0.36 b | 0.15a | 0.19b | 0.10 b | 28.34 | 6.69b |

Table S2 Properties of microbial co-occurrence network

| Microbial group | Bacteria |  |  | Fungi |  |  | All |  |  |
| --- | --- | --- | --- | --- | --- | --- | --- | --- | --- |
| The continuous cropping systems | Alfalfa | Corn | Sheepgrass | Alfalfa | Corn | Sheepgrass | Alfalfa | Corn | Sheepgrass |
| num.edges(L) | 839 | 488 | 197 | 166 | 166 | 289 | 1781 | 1033 | 522 |
| num.pos.edges | 456 | 263 | 105 | 143 | 99 | 194 | 1165 | 791 | 440 |
| num.neg.edges | 383 | 225 | 92 | 23 | 67 | 95 | 616 | 242 | 82 |
| ratio | 1.19 | 1.17 | 1.14 | 6.22 | 1.48 | 2.04 | 1.89 | 3.27 | 5.37 |
| num.vertices(n) | 94 | 98 | 95 | 96 | 94 | 96 | 804 | 570 | 498 |
| average.degree(Average K) | 17.85 | 9.96 | 4.15 | 3.46 | 3.53 | 6.02 | 4.43 | 3.63 | 2.10 |
| average.path.length | 2.02 | 2.44 | 2.99 | 3.56 | 3.26 | 3.68 | 4.53 | 3.92 | 1.42 |
| diameter | 6.22 | 7.47 | 6.94 | 8.85 | 8.20 | 10.21 | 14.10 | 11.38 | 6.60 |
| mean.clustering.coefficient(Average.CC) | 0.57 | 0.44 | 0.36 | 0.35 | 0.28 | 0.53 | 0.50 | 0.66 | 0.72 |


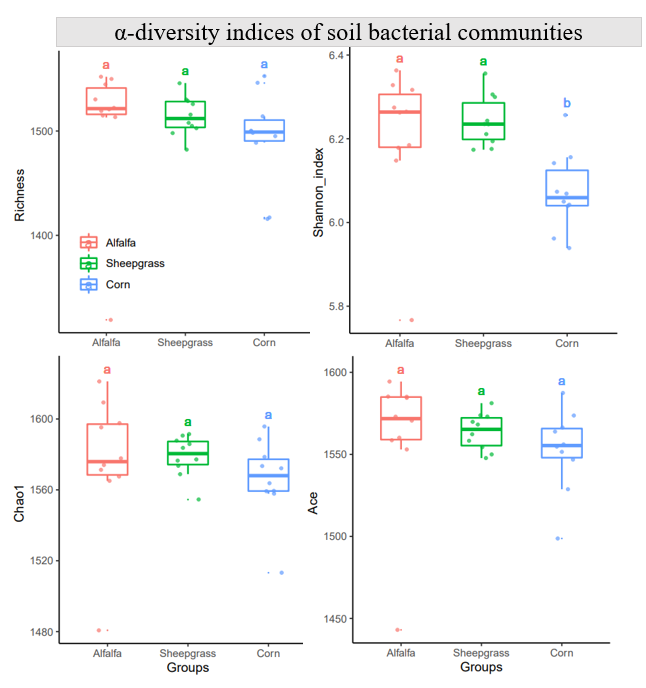


Figure S1. α-diversity of the soil bacterial communities in three systems. **a** Richness. **b** Shannon index. **c** Chao1. **d** ACE. Different letters on the column showed the significant difference among the three systems (Tukey’s test, p-value<0.05).


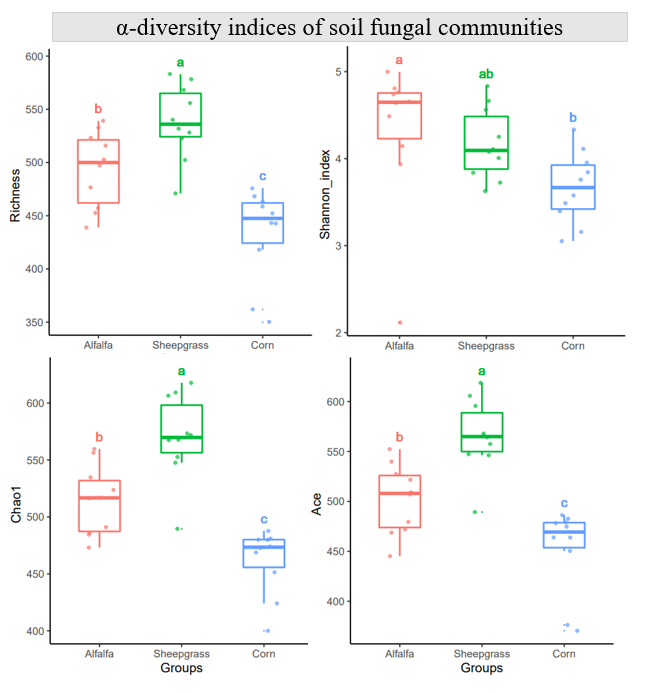


Figure S2. α-diversity of the soil fungal communities in three systems. **a** Richness. **b** Shannon index. **c** Chao1. **d** ACE. Different letters on the column showed the significant difference among the three systems (Tukey’s test, p-value<0.05).


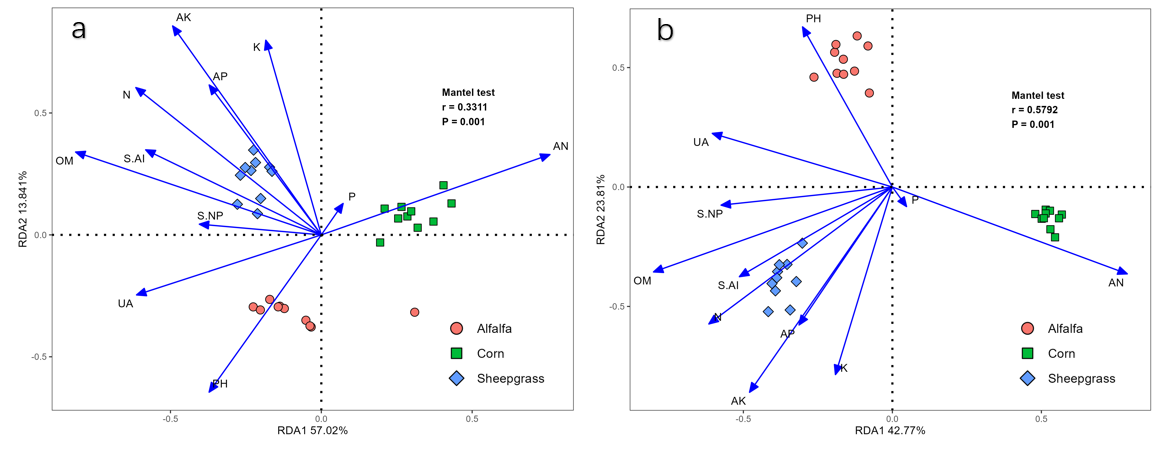


Figure S3. RDA ordination plots of soil bacterial (a) and fungal (b) communities under different continuous monoculture systems. Environmental variables are shown as blue arrows. Sample points are colored and shaped by treatment groups (corn, alfalfa, sheepgrass). Mantel test results (r and P values) are displayed in the upper right corner.


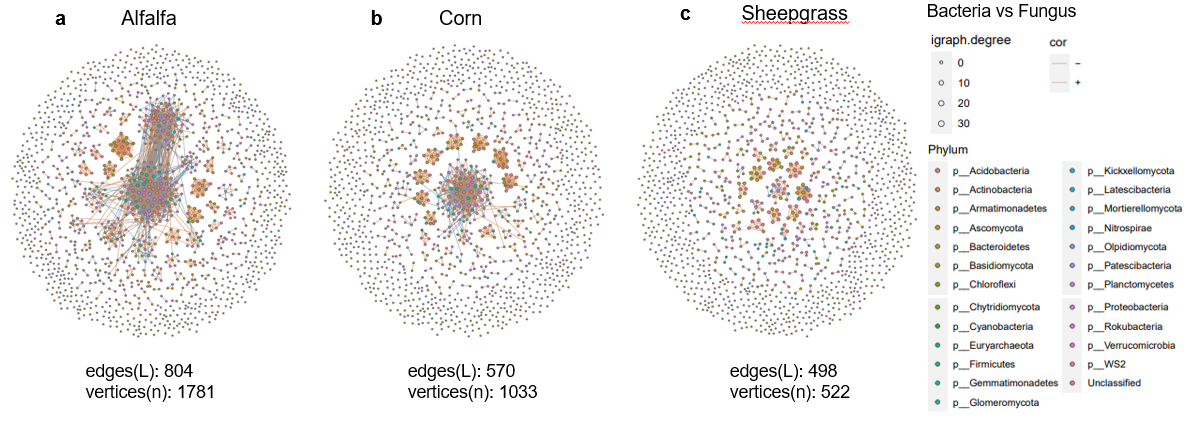


Figure S4. Associations across microbial groups (bacteria and fungus) differ among the continuous monoculture systems. Nodes are colored according to their taxonomic affiliation at phylum levels. The size of the nodes is proportional to the number of links per node. Link thickness is proportional to partial correlations between nodes and represents associative (blue, ρ>0.1) or exclusionary relationships (yellow, ρ<−0.1). The complexity of networks was estimated using network size (i.e. num.vertices (n), num.edges (L)). The lowercase letters in the upper left corner (in bold) are each network (a, b and c).
